# Personalized NanoMedicine: Towards new Theranostic Approach

**Published:** 2015-04-30

**Authors:** Madhavan Nair

**Affiliations:** Center of Personalized NanoMedicine, Institute of Neuroimmune Pharmacology, Department of Immunology, Herbert Wertheim College of Medicine, Florida International University, Miami, USA

Since decades, researchers are seeking novel therapeutic strategies to cure and monitor diseases for personalized health care needs. Numerous successful efforts are being made to design and develop personalized NanoMedicine approach to cure diseases at target site with minimum or no adverse effects. The designing of novel nanomaterials, maximum binding with therapeutic agent, controlled navigation, on demand or sustained release of drug, good biocompatibility, and maximum therapeutic potentials are crucial for effective theranostic approaches. Numerous studies have been conducted to formulate NanoMedicine and testing its efficacy using various in-vitro models. Unfortunately, such therapeutic nanoformulations are not well-tested yet using pre-clinical or human models.

Research group headed by Dr. Nair, at Center of Personalized NanoMedicine, Department of Immunology, Herbert Wertheim College of Medicine, Florida International University is actively engaged in exploring personalized NanoMedicine approach for target specific drug targeting to prevent neuroAIDS in drug using populations (Figure 1) [1-5]. Further the research group is also exploring sensing technologies to understand the effect of various drugs of abuse and psychological stress in HIV infected patients [6-7].

State-of-the-art, nano-engineered magnetic (Fe_3_O_4_) nanocarriers to deliver therapeutic cargo across blood brain barrier (BBB) to cure neuro-AIDS in drug addicts have been demonstrated [1-5]. To achieve on-demand site specific controlled release of drug, Prof. Nair also explored a second generation of magnetic nanoparticles called magneto-electro nanoparticles (MENPs) to cure Neuro-AIDS and cancer [1-2]. Presently, efforts are continuously being made at Dr. Nair’s laboratory to explore potentials of these NanoMedicine formulations to eradicate latent HIV in HIVE-SCID and drug addicted animal models. In summary, these studies lead to believe that there is a considerable scope to explore and develop novel nano-drug-carriers which are capable to deliver and to release desired therapeutics agent in sustained or controlled fashion across blood brain barrier (BBB) or any organ in the periphery without any side-effect.

Journal of Personalized NanoMedicine (JPNM) (http://jpnmjournal.org/aims_scope.php) is the official journal of the Society for Personalized NanoMedicine (http://s-pnm.org) and the purpose of this editorial is to describe the aims and scopes (http://jpnmjournal.org/aims_scope.php) of this journal and also to attract researchers for manuscript submission. This journal is an open on-line journal to all theoretical and experimental aspects related to Personalized NanoMedicine. JPNM invites original research articles, editorials, commentaries, prospective, case reports, technical notes, commercialization notes, reviews, mini-reviews, communications, and cutting edge-research synopsis to explore NanoMedicines for theranostics at personalized health care needs.

## Figures and Tables

**Figure 1:**
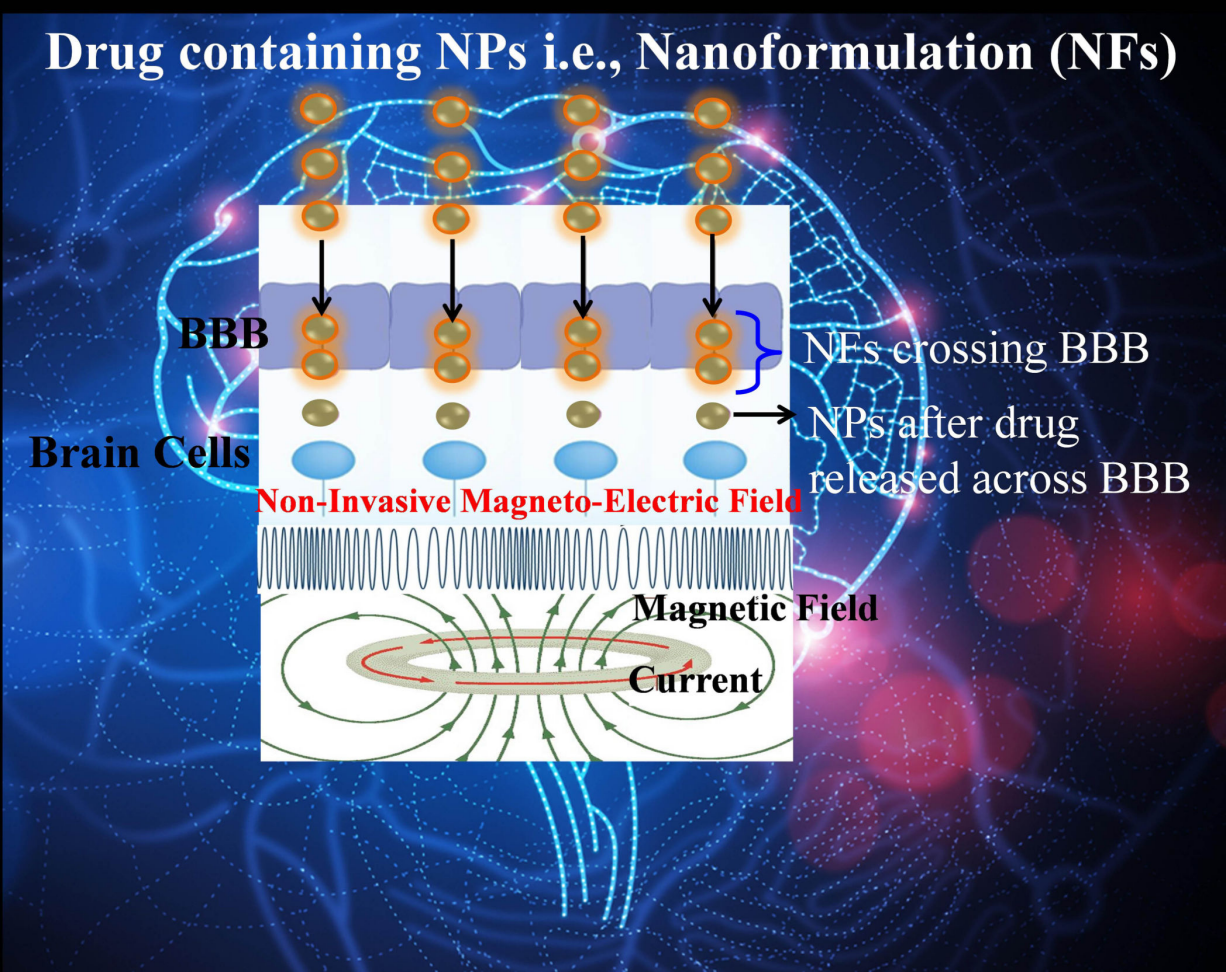
Illustration of an externally AC magnetic field stimulated blood brain barrier based drug delivery model.
